# Bilateral epidermoid vocal cysts. Case report

**DOI:** 10.1016/j.bjorl.2023.101357

**Published:** 2023-11-01

**Authors:** Regina Helena Garcia Martins, Claudia Mendonça Xavier, Alessandra Loli, Lucas Yamada Kurosaki, Elaine Lara Mendes Tavares, Andrea Joia Gramuglia

**Affiliations:** a Universidade Estadual Paulista “Júlio de Mesquita Filho” (Unesp), Faculdade de Medicina de Botucatu, Departamento de Oftalmologia, Otorrinolaringologia e Cirurgia de Cabeça e Pescoço, São Paulo, SP, Brazil; b Universidade Estadual Paulista “Júlio de Mesquita Filho” (Unesp), Faculdade de Medicina de Botucatu, São Paulo, SP, Brazil

## Introduction

Vocal cysts are classified as: retention (mucosal) or epidermic (composed of epithelial desquamation material). Vocal involvement depends on the volume of the cyst and its position. The voice is usually hoarse, instable, breathy and with lower pitch. We did not find reports of bilateral vocal cysts, especially in children or adolescents. Thus, the objective of the present study was to present a rare case of bilateral epidermoid vocal cyst in teenagers.

## Case report

**Figure 1:**
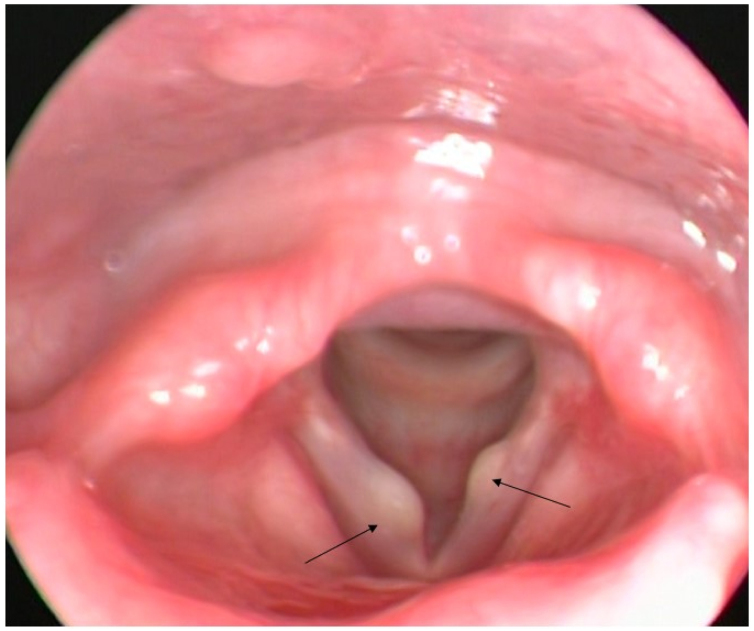
Bilateral vocal cyst.

Female, 14-years old, complained of hoarseness since childhood, with considerable worsening in recent months. Symptoms of weak voice, vocal fatigue and vocal instability were reported. A videolaryngoscopy was performed using a rigid telescope (8 mm in diameter, 70°, Asap brand, Germany), coupled to a multifunctional system video system (type XE-30, Eco X – TFT/USB – Germany) which identified two vocal cysts with yellowish content, one in each vocal fold, in different positions (Fig. 1) and an irregular glottic gap. At the videolaryngostroboscopy exam (using stroboscopic light, Atmos — Germany), the mucosal wave was absent over the lesions.

The analyzes of the vocal parameters indicated: GRBAS scale scores: G (3), R (3), B (2), A (1), S (2), maximum vowel phonation time (MPT)/a/ – 4 s. The acoustic analysis performed using the Multi-Dimensional Voice Program software (MDVP; KayPENTAX, USA), recorded: fundamental frequency (f0; 138.939), jitter % (14.032), Pitch Perturbation Coefficient (PPQ = 10.226), shimmer % (18.545), Amplitude Perturbation Coefficient (APQ = 13.181), Noise Harmonic ratio (NHR = 0.866) and Soft Phonation Index (SPI = 4.661).

The teenager underwent laryngeal microsurgery, using the bilateral lateral microflap technique, with significant and immediate voice improvement. The vocal parameters, one month after the surgery, were: G (1), R (1), B (0), A (0), S (0), MPT/a/ ‒ 11 s. Acoustic analysis: f0 (208.692), jitter % (0.661), PPQ (0.391), shimmer % (3.414), APQ (2.481), NHR (0.126) and SPI (4.954).

## Discussion

Bilateral epidermic vocal cysts are rare. They considerably compromise the voice, as shown in this clinical case. Bilateral cysts described in the literature are located in supraglottic regions such as aryepiglottic folds, vestibular region, or in valeculae.^1^, ^2^, ^3^ The rarity of bilateral epidermic vocal fold cysts was confirmed in a previous study in which we presented 72 cases of patients with vocal cysts, of which 26 were children, and all lesions were unilateral.^4^ In an equally expressive series, Kuranova et al.,^5^ presented 68 children with vocal cysts located in different locations (vestibular region, 22.1%; vocal folds, 22.1%; subglottic, 55.9%), all unilateral.

In this publication we demonstrate the severe vocal impairment caused by the presence of epidermic cysts in both vocal folds and the immediate benefits of microsurgery.

## Conclusion

Bilateral epidermic vocal cysts are extremely rare in the literature. We report the case of a teenager with bilateral vocal cysts with severe vocal impairment with complete recovery of his voice after surgery.

## Conflicts of interest

The authors declare no conflicts of interest.
